# Effect of robotic-assisted gait training on functional independence measure scores in patients with acquired brain injury: retrospective study

**DOI:** 10.3389/fresc.2025.1575148

**Published:** 2025-05-26

**Authors:** Andrea M. Ethier, Luis A. Escalante, Nathan West, Christina M. Kwasnica

**Affiliations:** Department of Neuro-Rehabilitation, Barrow Neurological Institute, St. Joseph’s Hospital and Medical Center, Phoenix, AZ, United States

**Keywords:** brain injury, exoskeleton, gait, robotics, therapy

## Abstract

**Introduction:**

Many individuals with acquired brain injury require inpatient rehabilitation services. Robotic devices, including robotic exoskeletons for gait training, have been shown to optimize rehabilitation efforts and functional outcomes. The purpose of this study was to investigate the effect of robotic-assisted gait training using the Ekso GT robotic-assisted gait training device (Ekso Bionics) on Functional Independence Measure (FIM) scores in patients with subacute acquired brain injuries.

**Methods:**

This retrospective study assessed patients who participated in traditional physical therapy during an acute neurological rehabilitation stay; study group participants also received at least 3 robotic-assisted gait training sessions during their rehabilitation stay. Patient medical records were reviewed retrospectively to collect patient demographic and clinical data, including patient age and sex, admission date, acquired brain injury category, number of robotic-assisted gait training sessions and session details, length of stay, and admission and discharge FIM overall scores, as well as the scores for the gait, transfer, motor, and cognition FIM subscales. Change in FIM score was used as the primary outcome measure.

**Results:**

The study group and the control group each included 56 patients (study group: 44 patients with cerebrovascular accident, 12 with traumatic brain injury; control group: 45 patients with cerebrovascular accident, 11 with traumatic brain injury). Summary statistics revealed similar characteristics between the groups in all areas except mean length of stay, which was significantly longer in the study group (*P* = 0.04) compared to the control group. Analysis of variance was used to assess the treatment effect between the study and control groups; no significant difference was found between the 2 groups in change in FIM scores between admission and discharge. Regression analysis showed a significant difference between the baseline and discharge scores in the study group for all 5 FIM categories assessed (overall, gait, transfer, motor, and cognition).

**Discussion/conclusion:**

These results indicate that robotic walking devices, when used for gait training in patients with acquired brain injury, led to similar improvements in FIM scores compared with traditional gait training. No negative effects were observed associated with the use of this robotic walking device in patients recovering from acquired brain injury.

## 1 Introduction

Acquired brain injuries (ABIs), including traumatic brain injuries (TBIs) and cerebrovascular accidents (CVAs), are among the leading causes of disability in the United States. According to the Centers for Disease Control and Prevention, about 3.2–5.3 million people in the United States are currently living with a TBI-related disability (1, 2). In addition, stroke is the leading cause of serious long-term disability in the United States, with more than 795,000 people in the United States experiencing a stroke each year (3). Two-thirds of these individuals require comprehensive, interdisciplinary therapy following the infarct (4). TBI and CVA often result in permanent limitations in mobility and cognitive abilities, which consequently can lead to the need for assistance from a caregiver to perform activities of daily living. TBI and CVA can be associated with lifelong health problems that may affect all aspects of a person's life and that have effects similar to the effects of a chronic disease (5). The economic burden of TBI and CVA and their long-term management are substantial. The lifetime economic cost of TBI, including direct and indirect medical costs, was estimated in 2006 to be approximately $76.5 billion (6). In 2017–2018, the total stroke-related costs in the United States were estimated to be almost $53 billion (3).

A large percentage of people who experience an ABI require inpatient rehabilitation. Typically, the goal of any inpatient rehabilitation facility is to improve patients’ independence and patients’ overall quality of life on a physical, emotional, and social basis. In short, the goal of inpatient rehabilitation is to return patients to the highest level of function possible. The Functional Independence Measure (FIM) was developed to assess patients’ disability and their progress during rehabilitation (7). The FIM is an 18-item, clinician-reported scale that assesses function and level of independence in 6 areas: self-care, continence, mobility, transfers, communication, and cognition. Each of the 18 items is graded on a scale of 1–7 based on level of independence; a score of 1 indicates that total assistance is required, and a score of 7 reflects complete independence. The FIM was designed to be sensitive to changes over the course of a patient's stay in an inpatient medical rehabilitation program.

Inpatient neurological rehabilitation stays have been getting shorter due to reductions in insurance reimbursements. Therefore, it has become imperative for inpatient rehabilitation therapists to choose optimally effective and efficient therapeutic tasks that will improve patients’ long-term functional mobility and level of independence, reflected in improved FIM scores. High-volume repetitions (8), high-intensity training (4), and specificity of training (9) are all known to result in improvements within the neurologically impaired population. However, completing these tasks with patients who are severely impaired and who require a high level of assistance can be challenging, and sometimes impossible. In addition, diminished activity tolerance for patients experiencing new neurological deficits can be a barrier to completing high-volume, high-intensity, task-specific practice.

Robotic devices, including robotic exoskeletons for gait training, are being used more widely in the rehabilitation setting as the field recognizes their potential to optimize rehabilitation efforts and functional outcomes. In many cases, robotic exoskeletons can allow patients to engage in high-volume step training, increase patients’ cardiovascular effort, and allow for earlier introduction of gait training. Traditional rehabilitation methods for gait training often require some form of body weight support for the patient, which puts a large demand on therapists (10, 11) and could result in job-related pain or injury (12). Robotic exoskeletons for gait training can help alleviate some of the physical demands on therapists (10) while offering high-quality stepping patterns and biomechanics (13). Compared to traditional gait training, robotic exoskeletons used for gait training have been shown to greatly increase the amount of work that can be done by impaired or nonfunctioning lower limbs (14).

Multiple studies have indicated that patients who receive robotic-assisted gait training after CVA are more likely to recover independent walking than patients who do not receive robotic-assisted gait training (15, 16). Some reports have suggested that the use of robotics in rehabilitation could result in improved motor skills that could transfer to other daily living domains that require similar skills (17). Although fewer studies support the use of robotic devices in TBI recovery, we concluded that the inclusion of patients with TBI would be appropriate for this study because both CVA and TBI are nonprogressive central nervous system conditions with similar impairments (18). Both patients with CVA and patients with TBI are likely to experience significant challenges in gait and mobility due to varying underlying causes, which may include muscle weakness or spasticity, impaired coordination, sensory and motor dysfunction, impaired functional endurance, and gait variability. The main goal of this study was to determine whether patients with ABI who received robotic-assisted gait training had a greater change in FIM scores than patients who did not receive robotic-assisted gait training.

## 2 Materials and methods

### 2.1 Study design and setting

We conducted a retrospective medical records review cohort study of patients with ABI, including TBI and CVA, who were inpatients from a single rehabilitation hospital between December 2014 and December 2016. Patients were required to receive a total of 3 hours of therapy a day, per the standard of care for a rehabilitation facility. This time was divided between physical, occupational, and speech therapy. In addition to their standard therapy, study participants also received gait training using the Ekso GT robotic walking device (Ekso Bionics) with a trained physical therapist, either as part of or in addition to their standard physical therapy time. Data from patients who received robotic-assisted gait training were compared against data from patients who received the standard of care only (i.e., without robotic-assisted gait training).

A power analysis using Power Analysis and Sample Size (PASS; NCSS, LLC; Kaysville, Utah) software determined cohorts of 70 patients were needed to achieve 90% power to detect a mean difference of an overall FIM score of 50 vs. 60, assuming a standard deviation of 18 in both groups and a significance threshold of 0.05.

This study was approved by the institutional review board (IRB) at St. Joseph's Hospital and Medical Center in Phoenix, Arizona, and conformed to the Declaration of Helsinki and the US Federal Policy for the Protection of Human Subjects. Due to the retrospective nature of this study, a waiver of authorization under the Health Insurance Portability and Accountability Act (1996) was submitted and approved by the IRB. Written informed consent was not required in accordance with the national legislation and institutional requirements.

### 2.2 Study participants

Patients who sustained either a TBI or a CVA and who were treated using the robotic walking device at least 3 times during their rehabilitation stay were included in this study. Patients were identified based on their use of the gait training device and were then filtered by a diagnosis of either TBI or CVA to determine their eligibility for study inclusion. Eligibility to use the Ekso GT at this rehabilitation facility included anthropometric measurements to ensure proper fit into the device; sufficient range of motion at the hips, knees, and ankles to endure movement sequencing of the device; ability to sustain the standing position for 20 minutes or more without adverse physiological events; ability to follow basic commands; lack of skin breakdown in areas where the device makes contact with the patient; and absence of fractures, pregnancy, or any other contraindication identified by the device's use guidelines. Patients were excluded from this study if they did not have a documented diagnosis of TBI or CVA or if they used the gait training device fewer than 3 times during their rehabilitation stay. A threshold of at least 3 sessions of robotic assisted gait training was set in our inclusion criteria to ensure adequate exposure to the intervention. We determined that one session would not be sufficient to demonstrate measurable changes in motor performance or gait function. Patients were included in the control group if they had sustained an ABI and met the criteria for the use of a robotic gait device; however, these patients did not actually use a robotic gait device. The control group data were obtained from patient records prior to their use of the robotic device on the rehabilitation unit.

### 2.3 Variables

Patients’ electronic medical records were reviewed retrospectively for patient age and sex, admission date, ABI category (TBI or CVA), number of gait training device sessions and session details, length of stay (LOS), and FIM scores at admission and discharge. Scores for 5 FIM categories were assessed at each timepoint: overall FIM score and scores for the FIM subcategories gait, transfer, motor, and cognition. The changes in FIM scores were the primary outcome measures for this study.

### 2.4 Data sources and measurements

All data presented in this study were collected from procedures that are part of the institutional standard of care; no further interventions or data collection instruments were implemented as a part of this study. The standard of care included determination of eligibility for the use of the robotic-assisted gait training device by each patient's primary physical therapist. After candidates were determined to be eligible, medical doctor clearance and orders were obtained prior to a formal device evaluation. Patients received education regarding the use of the robotic device as part of their plan of care and consented to the use of the device for their therapy. Device evaluations and sessions were completed by Ekso GT-certified physical therapists, each of whom completed formal training with the device prior to using it with patients. Data from each session were recorded in a paper chart; these data included session time, walking time, steps completed, and level of assistance provided by the device. The paper documentation was transcribed by the treating physical therapist into the patient's electronic medical record. All patients also received conventional physical therapy during their rehabilitation stay. Conventional therapy included at least 1 hour of physical therapy per day, 5 days per week, and could include strengthening exercises, balance and coordination training, range of motion exercises, functional training, pregait and gait training, and patient education with the focus on improving each patient's functional mobility and level of independence. Sessions with the gait training device could be part of the patient's required hours of therapy per day or could be conducted as “extra” therapy. This determination, as well as the total number of gait training device sessions completed, was patient-dependent and determined based on patient tolerance, availability of device-trained therapy staff, and the patient's LOS in the rehabilitation unit.

### 2.5 Ekso GT use protocol

Ekso GT is a wearable bionic suit that enables individuals with any degree of lower-extremity weakness to stand and walk with a natural, full-weight bearing, reciprocal gait. Sensors in the device detect shifts in the patient's weight and initiate steps. Battery-powered motors drive the legs forward in a stepping motion that mimics the gait pattern. This device is approved by the US Food and Drug Administration for use only in the clinical setting, with the supervision and assistance of certified physical therapists. Certification for use of the device includes multilevel training to ensure the competence of the trained therapist.

The device has different modes depending on the needs and abilities of each patient. For this study, the mode used was Pro Step Plus, in which steps are triggered by the patient's weight shift along with the initiation of forward leg movement. Weight shifts are completed by the patient independently or via facilitation of the treating therapist. In this mode, motor assistance for forward stepping can be programmed to be in an “adaptive” mode, where the device reads the efforts from the patient and dynamically adjusts to the patient's needs, or a “fixed” mode, where the treating therapist programs a certain threshold a patient must achieve for completion of a step (from 0 to 100, with a threshold of 100 indicating that the device completes the step at full assistance). If that threshold is not met by the patient, the device pauses for a predetermined time before completing the step for the patient. Each leg can be independently set to the level of assistance as deemed appropriate by the treating therapist. In addition, for patients in whom only one limb is affected, therapists can choose a mode in which motor assistance is turned off for the unaffected limb. Stance stabilization is provided within a programmed parameter, but forward stepping must be completed independently by the patient. Although the device has other modes, only Pro Step Plus with adaptive, fixed, and left- or right-limb-affected modes were used for patients who were included in this study.

### 2.6 Statistical analysis

Summary statistics were performed on baseline characteristics related to demographic and other treatment-related characteristics using means and standard deviations for continuous data and proportions and frequencies for discrete data. Analysis of covariance methods were applied to assess the treatment effect between study and control groups after adjusting for related covariates of age, LOS, and 5 FIM scores (overall FIM, gait, transfer, motor, and cognition). Within the study group, multivariable regression models were used to evaluate the association between the admission and discharge FIM scores after adjusting for the covariates age, LOS, number of sessions, and the impact of the number of Ekso sessions on discharge FIM scores.

## 3 Results

**Figure 1:**
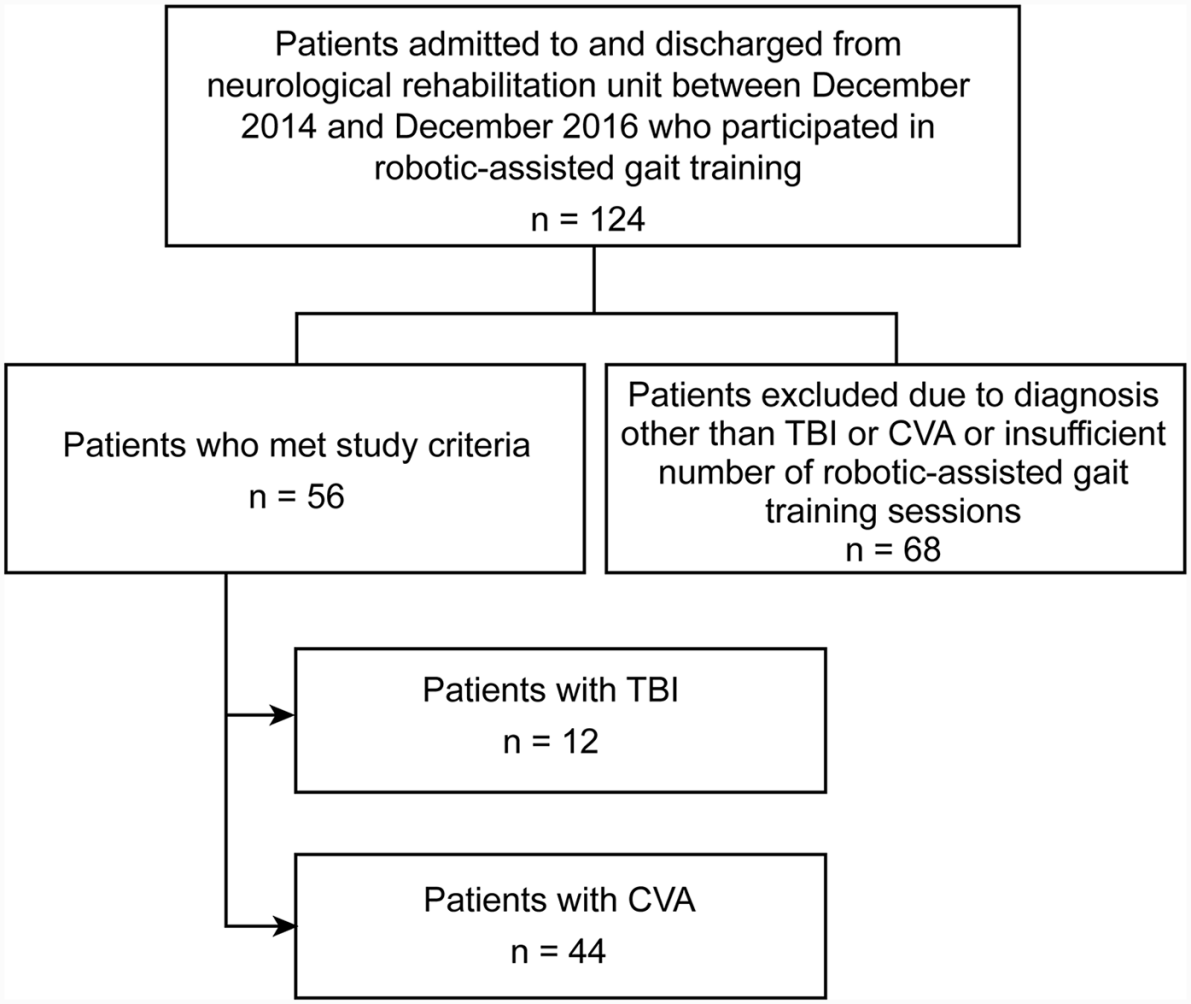
Flow diagram describing how patients were selected for inclusion to the study group. Of the 124 patients that we had data on, 56 met the study criteria, with 12 of those patients having a diagnosis of TBI and 44 of those patients having a diagnosis of CVA. Sixty-eight did not meet study criteria and therefore were excluded. Used with permission from Barrow Neurological Institute, Phoenix, Arizona.

In total, 124 patients received robotic-assisted gait training during the study period; of these, 56 patients were identified as appropriate for inclusion in this study. Forty-four had CVA, and 12 had TBI. A total of 68 patients were excluded from the study group due to not falling into the TBI or CVA diagnostic category or having an insufficient number of robotic-assisted gait training sessions (Figure 1). Fifty-six patients were included in the control group; 45 of these patients had CVA, and 11 had TBI.

### 3.1 Analysis of baseline characteristics

**Figure 2:**
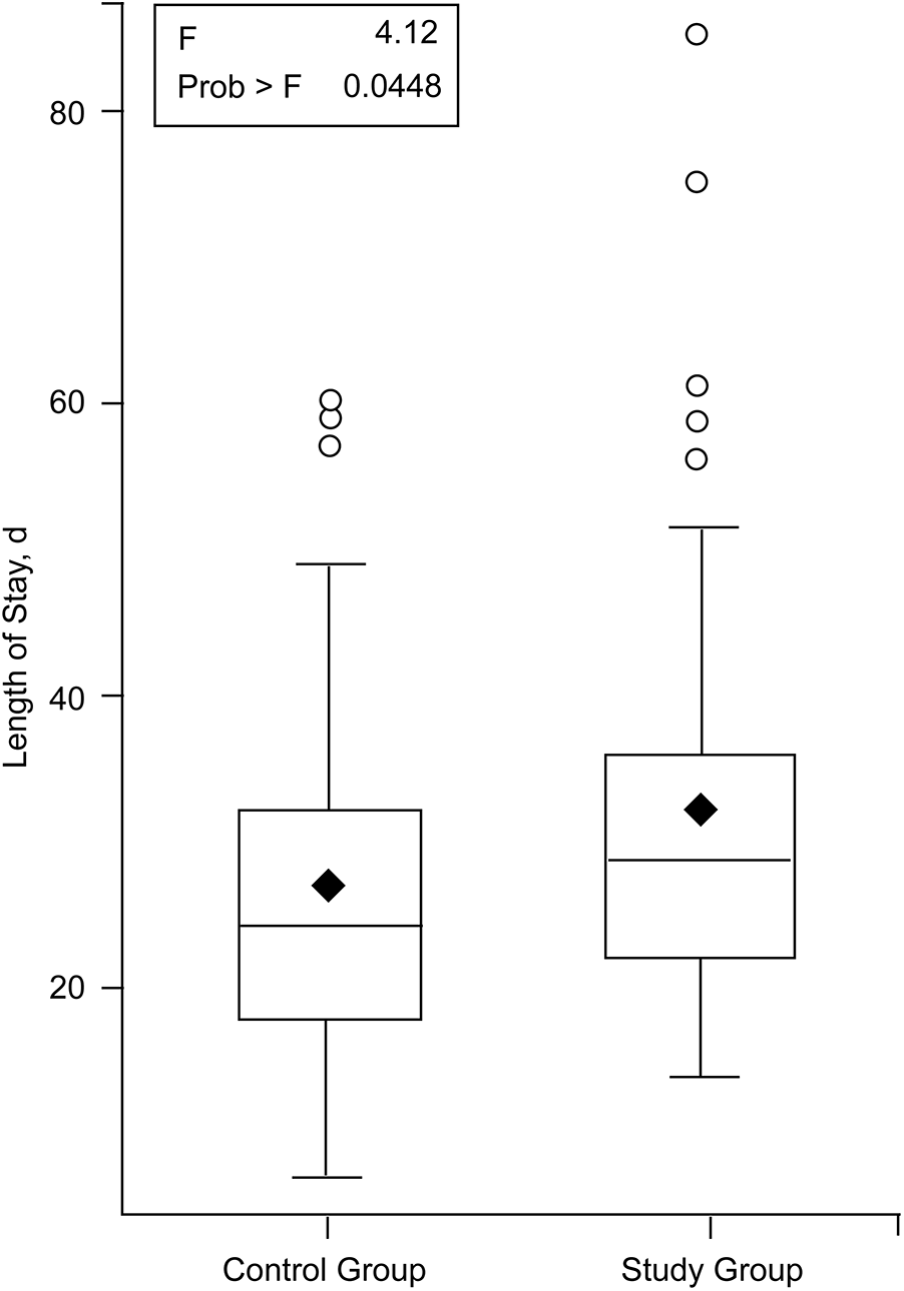
Length of stay comparison between study and control groups. The horizontal bar inside the boxes indicates the median, the diamond in the boxes indicates the mean, and the upper and lower ends of the boxes indicate the first and third quartiles. The whiskers indicate values within 1.5 times the interquartile range from the upper or lower quartile, and the circles indicate the data that are outliers. Used with permission from Barrow Neurological Institute, Phoenix, Arizona.

In the study group, 38 patients (68%) were men, and 18 patients (32%) were women. In the control group, 36 patients (64%) were men, and 20 patients (36%) were women. The mean (SD) patient age was 63.5 (16.6) years (range, 18–89 years) for the study group and 59.1 (19.0) years (range, 22–84) for the control group (*P* = 0.19). The mean (SD) LOS was 32.1 (14.5) days for the study group and 26.9 (12.5) days for the control group (*P* = 0.04) (Figure 2). The study group and control group had similar distributions for sex (*P* = 0.55) and diagnosis (*P* = 0.83).

### 3.2 Analysis of treatment comparisons

FIM scores were assessed for overall FIM as well as the FIM subscales for gait, transfer, motor, and cognition. The mean changes in FIM scores from admission to discharge were compared between the study and control groups, and no significant difference was found between the 2 groups in any category (Table 1). The mean (SD) overall change in FIM was 28.5 (13.1) for the study group and 24.8 (14.7) for the control group (*P* = 0.42). The mean change in FIM for gait was 2.02 (1.5) for the study group and 2.25 (1.31) for the control group (*P* = 0.06). The mean change in FIM for transfers was 1.86 (0.9) for the study group and 1.55 (1.11) for the control group (*P* = 0.17). The mean change in FIM for motor category was 20.1 (10.7) for the study group and 19.0 (11.31) for the control group (*P* = 0.97). The mean change in FIM for the cognition category was 6.05 (5.9) for the study group and 4.25 (4.66) for the control group (*P* = 0.13).

**Table 1:** Changes in Functional Independence Measure scores from admission to discharge.

FIM category	ΔFIM score		Standard error	*P*-value
	Study group	Control group		
Overall	28.5 (13.1)	24.80 (14.72)	2.62	0.43
Gait	2.02 (1.5)	2.25 (1.31)	0.28	0.06
Transfer	1.86 (0.9)	1.55 (1.11)	0.19	0.17
Motor	20.1 (10.7)	19.00 (11.31)	2.10	0.97
Cognition	6.05 (5.9)	4.25 (4.66)	0.87	0.13

Data are presented as mean (SD) unless otherwise indicated. FIM, Functional Independence Measure.

### 3.3 Regression analysis

Baseline FIM scores were predictive of discharge FIM scores for all models, including the following scores: the overall FIM (*P* = 0.001) as well as the subscales for gait (*P* = 0.03), transfer (*P* = 0.008), motor (*P* < 0.001), and cognition (*P* < 0.001) (Table 2). However, the number of Ekso sessions was not predictive of discharge FIM scores in any of the 5 models.

**Table 2:** Multivariate regression analysis predicting discharge Functional Independence Measure scores.

Model	Unstandardized beta coefficient	Standard error	95% confidence interval	*P*-value
Model #1 overall FIM				
Baseline FIM	0.87	0.13	0.60 to 1.14	<0.001
Age	−0.15	0.11	−0.37 to 0.07	0.176
Length of stay	−0.27	0.17	−0.62 to 0.08	0.127
Number of Ekso sessions	0.10	0.74	−1.37 to 1.58	0.889
Model #2 transfer				
Baseline transfer	0.55	0.20	0.15 to 0.96	0.008
Age	−0.02	0.01	−0.03 to 0.01	0.058
Length of stay	−0.03	0.01	−0.05 to −0.001	0.043
Number of Ekso sessions	0.01	0.05	−0.09 to 0.11	0.834
Model #3 gait				
Baseline gait	2.09	0.73	0.62 to 3.56	0.006
Age	−1.90	0.97	−3.85 to 0.04	0.055
Length of stay	−2.33	1.46	−5.26 to 0.60	0.116
Number of Ekso sessions	−0.34	6.48	−13.36 to 12.68	0.959
Model #4 motor				
Baseline motor	0.89	0.19	0.51 to 1.27	<0.001
Age	−0.13	0.09	−0.30 to 0.05	0.164
Length of stay	−0.21	0.14	−0.49 to 0.07	0.143
Number of Ekso sessions	0.06	0.60	−1.14 to 1.26	0.920
Model #5 cognition				
Baseline cognition	0.52	0.10	0.32 to 0.71	<0.001
Age	−0.01	0.04	−0.09 to 0.07	0.787
Length of stay	−0.10	0.06	−0.23 to 0.03	0.115
Number of Ekso sessions	−0.17	0.28	−0.73 to 0.39	0.539

## 4 Discussion

We conducted a comparative analysis between patients with ABIs who participated in robotic-assisted gait training and patients with ABIs who were treated with conventional physical therapy only and did not participate in robotic-assisted gait training. The 2 groups were similar in characteristics with the exception of mean LOS, which was significantly longer in the study group than in the control group. Patients who underwent robotic-assisted gait training received approximately 5 more days of therapy than those in the control group. However, in spite of the longer therapy time and the addition of robotic-assisted gait training, no significant improvement was seen in overall FIM scores.

We found similar admission-to-discharge changes in FIM scores between the groups in all areas of the FIM that we analyzed. Both the study group and the control group showed improvement in their functional scores from admission to discharge, but one group did not outperform the other. Despite the lack of statistical evidence shown in this study, we found no negative effect on a patient's FIM score change when a robotic-assisted gait device was incorporated into a patient's plan of care. Patients in both the study group and the control group demonstrated significant changes in all areas of the FIM. Therefore, it appears that the use of robotic-assisted gait training devices in patients with ABI is as effective in recovering walking ability in the rehabilitation setting as conventional or standard-of-care therapies. Our results are similar to those of other studies that have explored robotics for the recovery of gait ability in the stroke population, revealing no significant difference between traditional therapies and robotic-assisted gait training (19, 20). As previously stated, high-volume, high-repetition exercises, also known as massed practice, yield improved results in functional gains in neurologically impaired patients (21). Because this theory has been generally accepted in the rehabilitation setting, the standard of care also aims to achieve high-volume, high-repetition efforts in traditional gait training. Therefore, it could be suggested that a standard of care that uses these theories as their framework could actually mirror the high volume, large repetitions that are achieved in robotic-assisted gait training. Future studies that quantify the volume of traditional gait training compared to robotic-assisted gait training could further investigate this theory, as well as investigating the cardiovascular effort required for each type of gait training.

Although no significant differences were found in mean changes in FIM scores between the robotic-assisted gait training group and the traditional gait training group, a closer examination of variability in improvements revealed subtle differences in stability. The overall variability in FIM scores as shown by the SDs was comparable between groups (study group SD = 13.1 vs. control group SD = 14.7), indicating similar overall stability in functional improvements. However, when specific categories were analyzed, the study group showed less variability in transfer related gains compared to the control group (study group SD = 0.9 vs. control group SD = 1.1), suggesting more predictable outcomes in structured tasks such as transfers. In contrast, the gait category exhibited higher variability in the study group (study group SD = 1.5 vs. control group SD = 1.3), which may reflect differences in patient responses to robotic intervention. Despite these differences, both training methods demonstrated similar levels of improvements and stability across subscales, reinforcing the reliability of functional outcomes in both groups.

Regression analysis indicates a significant correlation between admission FIM scores and discharge FIM scores among study group participants. However, our comparison analysis between the control group and the study group indicates that this improvement was not significantly affected by the use of the gait training device or the number of sessions completed. This finding supports a previous study that concludes that functional status at the time of admission to a rehabilitation hospital is a primary predictor of outcomes at the time of discharge (22). This finding does not change based on the number of robotic-assisted gait training sessions that were completed.

Another area of interest that could be explored in future studies is patient- and therapist-perceived improvements in strength, endurance, and gait quality with the use of robotic-assisted gait-training devices. In our clinical experience, patients whose therapy is supplemented with robotic assistance often subjectively report positive improvements in these areas. It would also be interesting to explore depression scales and overall reports of mood for those whose therapy includes robotic-assisted gait training devices compared to those whose therapy does not include them.

### 4.1 Study limitations

Previous studies on the use of robotic walking devices have generally been limited by small sample sizes (23), and this study is no exception. Selection criteria limited the number of patients who were eligible for study inclusion, which resulted in a smaller sample size than initially anticipated. In addition, the retrospective nature of the study meant that no follow-up data were available. Future prospective studies should be designed to allow for the collection of long-term outcomes after the use of robotic walking devices and should use a larger sample size, as suggested by our power calculation.

The FIM is a broad measure used to determine a patient's level of disability and how much assistance they require to carry out activities of daily living. However, it does not quantify specific gait characteristics or balance abilities. System uncertainties, including variations in patient dynamics, unpredictable forces, or interaction forces between the patient and the robot, were not accounted for, nor can they be tracked by FIM scores. The use of more specific gait and balance outcome measures may better identify areas of improvement that are not detectable on the FIM. In addition, given the retrospective nature of our study, no single individual was consistently responsible for FIM scoring—instead, several therapists and nurses contributed to the scoring of individuals in both groups, which could create an issue with interrater reliability. The FIM has been found to have good interrater reliability for the overall score, but it has been shown to be poor when scoring items concerned with assessing independence in walking (24).

Another limitation of this study was the significant difference in LOS between the groups. Patients in the study group had a statistically significant longer LOS than those in the control group. In addition, patients in the study group often received robotic-assisted gait training as an adjunct to standard-of-care therapy. However, changes in FIM scores were not statistically significant between groups, and FIM scores were not better in the study group patients despite the increase in the amount of therapy they received.

Finally, robotics, especially in the realm of neurological rehabilitation, has seen substantial progress over the past few years. Given the data collection period (between 2014 and 2016), these advancements may affect how applicable the study's findings are to current devices or protocols. As such, there may be differences in outcomes when newer devices and protocols are employed. Further research is necessary to determine whether the earlier findings still apply with the newer, more advanced robotic systems available today.

## 5 Conclusions

The use of robotic technology is rapidly becoming more prevalent in rehabilitation settings. This study demonstrated that robotic walking devices used for gait training in patients with ABI led to similar improvements in FIM scores compared to patients who received traditional gait training. Although we cannot conclude that robotic walking devices yield greater FIM score changes compared to traditional therapies, we can highlight the fact that no negative effect was associated with the use of robotic-assisted gait training devices in patients recovering from ABI. These devices can be used to work toward the goal of improving patients’ functional outcomes and may help alleviate therapist strain. Further research is warranted to identify other benefits of use within the inpatient rehabilitation setting, including patient and therapist perceptions of its use and the effects of its use on overall patient mood. In addition, it would be useful to compare patient outcomes across different types of robotic-assisted gait training devices to assess their effectiveness in relation to one another. The addition of gait-specific measures may also be helpful in determining the benefits of using robotic-assisted gait training devices when addressing specific functional impairments and reducing compensatory strategies in patients recovering from ABI.

## Data Availability

The raw data supporting the conclusions of this article will be made available by the authors, without undue reservation.
